# Feasibility of internet-delivered cognitive-behavior therapy for obsessive-compulsive disorder in youth with autism spectrum disorder: A clinical benchmark study

**DOI:** 10.1016/j.invent.2022.100520

**Published:** 2022-03-04

**Authors:** Frida Wickberg, Fabian Lenhard, Kristina Aspvall, Eva Serlachius, Per Andrén, Fred Johansson, Maria Silverberg-Mörse, David Mataix-Cols

**Affiliations:** a Stockholm Health Care Services, Region Stockholm, Sweden; b Centre for Psychiatry Research, Department of Clinical Neuroscience, Karolinska Institutet, & Stockholm Health Care Services, Region Stockholm, Sweden; c Child and Adolescent Psychiatry, Department of Clinical Sciences, Lund University Lund, Sweden; d Sophiahemmet University, Department of Health Promotion Science, Stockholm, Sweden

**Keywords:** Autism, Obsessive-compulsive disorder, Youth, Treatment, Internet

## Abstract

Obsessive-compulsive disorder (OCD) is a treatable condition that often requires specialist care, particularly when comorbid with autism spectrum disorder (ASD). However, specialist clinics are few and typically located in large medical centers. To increase availability of evidence-based treatment for OCD in individuals with ASD, we adapted an internet-delivered cognitive behavior therapy (ICBT) protocol to suit the needs of these individuals and conducted a feasibility study (*N* = 22). The primary outcome was the clinician-rated Children's Yale-Brown Obsessive-Compulsive Scale (CY-BOCS), administered at pre- and post-treatment as well as 3 months after treatment. ICBT was deemed acceptable and was associated with clinically significant improvements in CY-BOCS scores, corresponding to a large within-group effect size (Cohen's *d* = 1.33). Similarly, significant improvements were observed in most of the secondary parent- and self-rated measures. Approximately 60% of the participants were classed as treatment responders and 50% were in remission from their OCD at the 3-month follow-up. To provide a meaningful benchmark, we also analyzed data from a specialist clinic that regularly treats individuals with comorbid OCD and ASD (*N* = 52). These analyses indicated that specialized in-person CBT produced significantly larger effects (*d* = 2.69) while being markedly more resource demanding, compared to ICBT. To conclude, ICBT can be successfully adapted to treat OCD in youth with ASD and may be a viable alternative for those who do not have direct access to highly specialized treatment. Further improvements of the treatment protocol based on participant and therapist feedback are warranted, as is a formal test of its efficacy and cost-effectiveness in a randomized controlled trial.

## Highlights

• Obsessive-Compulsive Disorder (OCD) is a frequent comorbidity in Autism Spectrum Disorder (ASD), causing impairment and suffering.
• We developed an internet-delivered cognitive behavior therapy intervention (ICBT) to treat OCD in youth with ASD.
• We benchmarked the new ICBT intervention with gold standard CBT treatment at a specialized clinic.
• This first feasibility study provided encouraging results regarding clinical efficacy, feasibility and scalability.
• Further development of the intervention and rigorous testing in well-controlled clinical trials are warranted.

## 1 Introduction

Autism spectrum disorders (ASD) affect about 2% of all children and adults (Centers for Disease Control and Prevention, 2021) and are characterized by difficulties with communication and social interaction as well as restricted interests and repetitive behaviors. Obsessive-compulsive disorder (OCD) is one of the most common psychiatric comorbidities in young people and adults with ASD. Obsessions and compulsions must be carefully differentiated from repetitive behaviors or special interests that are typical of autism (Russell et al., 2005). OCD symptoms are experienced as intrusive, distressing and time-consuming (Russell et al., 2005). About 10% to 20% of individuals with ASD also have OCD (Neil and Sturmey, 2014), compared to about 1.3% in the general population (Kosidou et al., 2017; Fawcett et al., 2020).

Obsessive-compulsive disorder (OCD) is a treatable condition that often requires specialist care, particularly when comorbid with ASD. However, specialist clinics are few and typically located in large medical centers. Cognitive behavior therapy (CBT) is recommended as the first-line treatment for OCD (American Psychiatric Association, 2007). When appropriately adapted and delivered by specialist clinicians, CBT can be effective for OCD in autistic individuals (Kose et al., 2018; Flygare et al., 2020; Martin et al., 2020), though the reported effects are somewhat smaller compared to those reported in non-autistic individuals with OCD (Murray et al., 2015; Jassi et al., 2021). For example, in the largest naturalistic study to date, 53% of 172 children with OCD and ASD were classed as treatment responders, compared to nearly 77% of non-autistic children with OCD (Jassi et al., 2021).

Internet-delivered CBT (ICBT) developed as a method to overcome common treatment barriers, such as shortage of trained therapists, geographical distances to specialist clinics and socioeconomic inequalities. In essence, ICBT mimics traditional in-person CBT in terms of content, the only difference being the mode of delivery (Hedman et al., 2012). The safety, efficacy, durability and cost-effectiveness of ICBT for children and adolescents for OCD have been well established (Aspvall et al., 2018; Lenhard and Andersson, 2017; Lenhard et al., 2014; Aspvall et al., 2021; Lenhard et al., 2020; Lenhard et al., 2017). A recent study demonstrated non-inferiority of ICBT compared to in-person CBT for OCD when delivered in a stepped-care fashion (Aspvall and Andersson, 2021). However, all previous studies systematically excluded young people with ASD, rendering the generalization of the results to this particular population uncertain.

There are several potential advantages with an internet-based treatment for individuals with ASD. Digital communication is perceived as a more comfortable way of communication for individuals with ASD (Benford and Standen, 2009) and it provides a high degree of visual support and structure, features that have been suggested to be helpful for children and adolescents with ASD (Scarpa et al., 2016).

This study aimed to evaluate the feasibility of ICBT for OCD in youth with ASD. We hypothesized that ICBT would be a feasible and acceptable treatment format for young people with OCD and ASD. For benchmarking purposes, we evaluated the outcomes of a separate group of youth with OCD and ASD who were treated with in-person CBT at a specialized OCD and related disorders clinic. The results from this study will help to further optimize the treatment protocol and be the basis of a power calculation for a definitive trial.

## 2 Material and methods

### 2.1 Study design

We conducted an open feasibility study evaluating a 16-week, ASD-adapted ICBT intervention for OCD in young people with ASD. This study was approved by the Swedish Ethical Review Authority (2017/2460-31) and pre-registered at ClinicalTrials.gov (NCT03473080).

For benchmarking purposes, we analyzed the clinical outcomes of a separate group of young people with OCD and ASD who were treated consecutively in our specialist OCD and related disorders clinic and signed an informed consent form. The clinic data collection was also approved by the Swedish Ethical Review Authority (2015–1977-31/4).

Patients in the two groups were selected based on similar inclusion criteria (see participants section) and were assessed by the same clinical team. For both groups, measurement points were pre-treatment, post-treatment and 3-month follow-up.

### 2.2 Participants

Study participants were recruited, assessed and treated at a specialized outpatient OCD and related disorders clinic within the regional Child and Adolescent Mental Health Service (CAMHS) in Stockholm, Sweden. The clinic receives referrals primarily from Region Stockholm but also from the rest of the country and offers specialized multidisciplinary care to children and adolescents with OCD and related disorders. The multidisciplinary team has extensive experience assessing and treating children with comorbid OCD and ASD. Currently, approximately 30% of all referrals of children with OCD also have a documented ASD diagnosis. The assessment includes careful differential diagnosis and differentiation between repetitive behaviors that are typical of autism and genuine obsessions and compulsions. All participants and their legal guardians provided informed consent prior to participation in the study.

#### 2.2.1 ICBT sample

Participants were eligible for inclusion if they fulfilled the following criteria: a diagnosis of OCD according to the Diagnostic and Statistical Manual of Mental Disorders, Fifth Edition (DSM-5; American Psychiatric Association, 2013), a DSM-5 diagnosis of ASD (confirmed by medical records), a total score of ≥16 on the Children's Yale-Brown Obsessive-Compulsive Scale (CY-BOCS; Scahill et al., 1997), age between 7 and 17 years, ability to read and write Swedish, access to the internet, a parent able to co-participate in the treatment and for patients on psychotropic medication: a stable dose for the last 6 weeks prior to pre-treatment assessment. Individuals were excluded if any of the following criteria were met: psychosis, bipolar disorder, severe eating disorder, suicidal ideation, intellectual disability, not able to read or understand the basics of the ICBT material, completed CBT for OCD within last 12 months (defined as at least 5 sessions of CBT including exposure and response prevention [ERP]) and ongoing psychological treatment for OCD or any anxiety disorder.

#### 2.2.2 Benchmark sample

Participants were consecutive referrals who were regularly treated at our specialist OCD and related disorders clinic, had a DSM-5 diagnosis of OCD, a comorbid DSM-5 diagnosis of ASD (confirmed in the medical records), a total score of ≥16 on the CY-BOCS, age between 7 and 17 years and had available data at pre-treatment, post-treatment and 3-month follow-up. Patients with a diagnosis of intellectual disability were excluded. As this was a naturalistic sample, participants on psychotropic medication or whose medication changed during the treatment period were not excluded.

### 2.3 Measures

Unless otherwise stated, all measures were administered at pre-treatment, post-treatment and 3-month follow-up. The primary outcome measure was the CY-BOCS, a semi-structured clinician-administered rating scale measuring OCD symptom severity in children and adolescents. The CY-BOCS has demonstrated high internal consistency and good to excellent interrater agreement for subscale and total scores (Scahill et al., 1997). In our sample Cronbach's alpha was α = 0.76. The Clinical Global Impression – Severity scale (CGI-S) is a brief clinician rating of symptom severity (Guy, 1976). The Clinical Global Impression – Improvement scale (CGI-I) is a brief clinician rating of symptom severity change, used to determine improvement (Guy, 1976). The clinician-rated Internet Intervention Patient Adherence Scale (iiPAS) was used in the ICBT group as a brief clinician-rated measure of adherence to internet-delivered interventions. The iiPAS has demonstrated robust psychometric properties and associations with objective measures of ICBT patient adherence (Lenhard et al., 2019). The Obsessional Compulsive Inventory – Child version (OCI-CV) is a self-rated measure of OCD symptom severity. The OCI-CV is strongly and significantly correlated with clinician-rated OCD symptom severity and parent and child reports of impairment related to OCD (Foa et al., 2002). The Family Accommodation Scale – Self Rated (FAS-SR) is a 12 item parent-report scale measuring parental accommodation of OCD symptoms, with good internal consistency, convergent and discriminant validity (Flessner et al., 2009). The Work and Social Adjustment Scale – Child and Parent Version (WSAS-Y C/P) is a 5 item self- and parent-rating scale of general functioning. The WSAS-Y has demonstrated excellent internal consistency across diagnostic groups and time-points, adequate test–retest reliability and strong convergent validity with other measures of functional impairment, as well as sensitivity to change (Jassi et al., 2020). Depressive symptoms were assessed using the short version of the Mood and Feelings Questionnaire, Child- and Parent version (SMFQ-C/P). The SMFQ-C/P has shown to be a reliable instrument for the discrimination of depressed and non-depressed youth (Angold et al., 1996). The Client Satisfaction Questionnaire (CSQ-8) was used to assess participants' satisfaction with treatment at post-treatment only. The maximum score on the CSQ is 32 points, with higher points indicating more satisfaction. The CSQ-8 is validated as a reliable measure of global patient satisfaction and is strongly associated with patients' adherence to treatment and clinical outcomes (Attkisson and Zwick, 1982). The Treatment Credibility and Expectancy Scale (TCES) was administered for the ICBT sample as a measure of treatment credibility after two weeks of treatment. The TCES has demonstrated high internal consistency and good test-retest reliability (Borkovec and Nau, 1972).

### 2.4 Patient and clinician feedback

Patient and clinician feedback regarding the feasibility of the ICBT program was collected. Free text questions were asked throughout the ICBT treatment and patients were asked about their experiences with the treatment as well as for suggestions for improvement. Open verbal feedback was obtained from the families at the post-treatment assessment. We also ran a focus group meeting with the six ICBT therapists in the study to gather their experiences and their suggestions for improvement. All feedback was documented and summarized by the first author (FW) and two master-grade psychologists.

### 2.5 Procedure

#### 2.5.1 ICBT sample

The study was advertised at the clinic's website and in local newspapers. For self-referral, an initial screening telephone interview was conducted with a parent to assess eligibility. If no exclusion criteria were fulfilled at this stage, an in-person appointment for psychiatric assessment was scheduled. Participants that were referred to the clinic from local outpatient CAMHS services in Stockholm and other parts of Sweden all underwent a psychiatric assessment as part of the standard care procedure. Families living far from the clinic were offered a video call as an alternative to the in-person appointment.

The psychiatric assessment included administration of the Mini International Neuropsychiatric Interview for Children and Adolescents (MINI-KID) (Sheehan et al., 1998), to verify diagnostic criteria of OCD and comorbidity, the CY-BOCS as well as CGI-S to assess OCD symptom severity. The diagnosis of ASD was confirmed by previous medical records, as ASD was formally assessed by the primary and secondary services prior to a referral, according to local practice guidelines. Patients who fulfilled all inclusion and no exclusion criteria were provided with written and verbal study information and offered participation. Self- and parent-reported measures were administered online at all time points. ICBT treatment started within two weeks after inclusion.

#### 2.5.2 Benchmark sample

All patients in the benchmark sample were referred to our specialist OCD clinic from local outpatient CAMHS services in Stockholm and other parts of Sweden. After referral, an initial assessment was conducted at the clinic, or in some cases by video call. As with the ICBT group, the psychiatric assessment included administration of the MINI-KID (Sheehan et al., 1998), the CY-BOCS and the CGI—S. The diagnosis of ASD was confirmed in the electronic medical records. After the CBT treatment, post-treatment measures were administered, including clinician-rated measures at the clinic and self- and parent-rated online measures. The same procedure was repeated at the 3-month follow-up. Changes in psychotropic medication and additional psychological treatment were monitored and documented throughout the study period.

### 2.6 Interventions

#### 2.6.1 ICBT

The digital intervention was an adaptation of our previously developed ICBT program for non-autistic young people with OCD (Lenhard et al., 2014; Lenhard and Andersson, 2017; Aspvall et al., 2018; Aspvall and Andersson, 2021). The adapted intervention consisted of 14 modules covering psychoeducation about OCD and ASD, including information on how to differentiate OCD symptoms from ASD-typical repetitive behaviors (modules 1–3), ERP (modules 4–13) and relapse prevention (module 14). Throughout the treatment, specific additional information and exercises related to ASD were given. The purpose of these ASD adaptations was not to treat the core symptoms of ASD but to enable ERP for the OCD symptoms. This included information on how to use visual support and time aids, regulate strong feelings, facilitate generalization of exposure exercises and reduce mental rigidity during exposure tasks. High parental involvement was encouraged throughout the treatment.

Children and their parents used separate accounts to log in to the secure online platform where they took part of reading materials, videos, animations, illustrations and exercises. There were two age-appropriate versions of the program, one for children aged 7–12 and one for adolescents aged 13–17 (see Aspvall and Andersson, 2021 for details). Parents and children followed the same structure in their respective parts of treatment. The parent protocol also included focus on family accommodation, parental coping strategies and how to coach their child through treatment.

The supporting online therapists were licensed psychologists trained to treat OCD. Therapist contact was provided asynchronously through written messages in the online platform or, when necessary, through telephone calls (see Aspvall and Andersson, 2021 for details).

#### 2.6.2 In-person CBT (benchmark sample)

The in-person CBT intervention was manualized and based on validated pediatric OCD treatment protocols (March et al., 1994) and specifically adapted for young people with ASD. The program consisted of a standard CBT protocol for OCD including education about OCD and treatment rationale, goal formulation, therapist-guided ERP, maintenance of treatment gains and relapse prevention. Homework exercises between sessions were strongly encouraged. In addition to this, this adapted program also included specific strategies to facilitate the young autistic person's engagement with ERP. These strategies included usage of time aids and visual support, adjusting inappropriate rituals to functional routines, setting up schedules for daily activities and for participation in exposure exercises and the introduction of new rules that went in line with the treatment goals. Parents also participated in an OCD course, partially focusing on parent behaviors and family accommodation.

The standard arrangement for the intervention was 24 h of treatment over a period of ten weeks, with a one-hour session and a three-hour session per week during the first six weeks and a one-hour session per week during the following four weeks. Two licensed psychologists were normally involved in each treatment, usually with both psychologists participating every other session and one of the psychologists in the other sessions. Sessions were conducted at the clinic, in the patient's home or in public spaces, according to individual needs.

### 2.7 Statistical analysis

Due to the uncontrolled nature of the study design, our a priori analytic strategy was to conduct separate within-group analyses for the ICBT and benchmark samples, without formal comparisons between the groups. Thus, improvement on the primary outcome measure (CY-BOCS) was analyzed using mixed effects models with fixed effects for time and random effects for intercept and subjects over time (i.e., random slope model) separately in each of the groups. Mixed effects modelling is an appropriate way to handle missing data (Gueorguieva and Krystal, 2004). Corresponding within-group effect sizes were estimated using Cohen's *d,* with a value of 0.8 or larger denoting a large effect (Cohen, 1988).

Treatment response was defined as a CGI-I rating of 1 – “Very much improved” or 2 – “Much improved” and a CY-BOCS total score reduction of ≥35%. Remission was defined as a CGI-S of 1 – “Normal, not at all ill” or 2 – “Borderline mentally ill” and a CY-BOCS total score of 12 or below (Mataix-Cols et al., 2016).

In a post hoc analysis, we followed the method outlined by Minami et al. (Minami et al., 2008), to benchmark the two intervention groups on their respective effect sizes at the 3-month follow-up, using the recommended effect size difference of Δ = 0.2 (i.e., 1/5 of a standard deviation) as the maximum difference for claiming clinical equivalence. The statistical significance for this difference was tested using a noncentral t distribution (Minami et al., 2008). This post hoc analysis was only done for the primary outcome measure (CY-BOCS).

Paired within-group significance testing of dichotomous outcomes was done with McNemar tests. Indicators of feasibility were patient satisfaction ratings, patient adherence ratings and number of completed sessions or modules.

## 3 Results

### 3.1 Sample characteristics

**Fig. 1:**
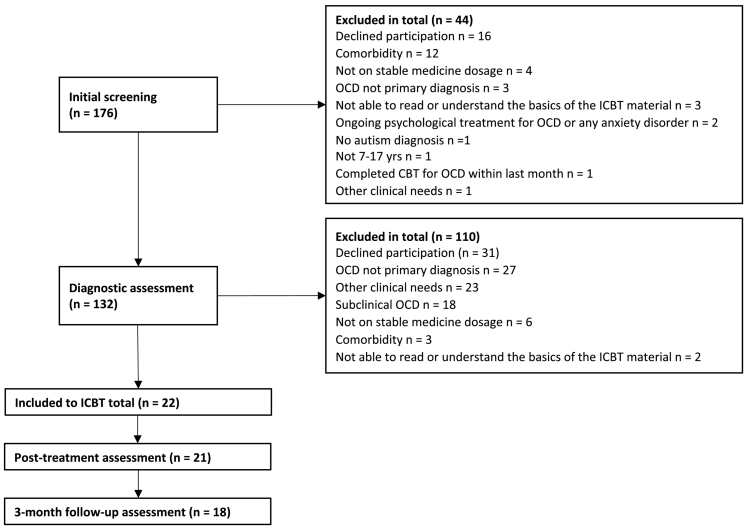
ICBT sample flow chart.

Out of 176 individuals screened for participation in the ICBT sample, 22 met inclusion criteria and were included in the study (Figure 1). The benchmark sample consisted of 54 individuals treated at our clinic and meeting the pre-specified inclusion criteria. The socio-demographic and clinical characteristics of both samples are presented in Table 1. The two samples were largely comparable, but the benchmark sample was somewhat more severe at baseline (23.8 vs 21.7 points on the C-YBOCS).

**Table 1:** Sample characteristics pre-treatment.

		ICBT	Benchmark	p
N		22	54	
Demographics				
Sex, n (%)	Girl	7 (33%)	21 (39%)	n.s.
	Boy	13 (62%)	32 (59%)	
	Other	1 (5%)	1 (2%)	
Age, mean (SD)		13.9 (1.6)	14.3 (2.4)	n.s.
Parental education, n (%)	Primary <9 yrs	1 (5%)	0 (0%)	n.s.
	Primary ≥9 yrs	1 (5%)	7 (13%)	
	High school	7 (33%)	10 (19%)	
	University <2 yrs	3 (14%)	8 (15%)	
	University ≥2 yrs	9 (43%)	22 (42%)	
	Post-graduate	0 (0%)	3 (6%)	
	Other	0 (0%)	3 (6%)	
Clinical severity				
CY-BOCS total score, mean (SD)		21.7 (3.7)	23.8 (3.5)	0.02
CGI-S, mean (SD)		4.0 (0.4)	4.4 (0.6)	0.005
Comorbidity				n.s.
Depression, n (%)		0 (0%)	11 (20%)	
ADHD, n (%)		11 (52%)	28 (52%)	
Anxiety disorder, n (%)		2 (10%)	5 (9%)	
Eating disorder, n (%)		0 (0%)	1 (2%)	
Tic disorder, n (%)		2 (10%)	0 (0%)	
BDD, n (%)		1 (5%)	0 (0%)	
Excoriation disorder, n (%)		1 (5%)	0 (0%)	
Number of comorbid diagnoses, n (%)	0	9 (41%)	18 (33%)	
	1	9 (41%)	28 (52%)	
	2	4 (18%)	7 (13%)	
	3	0 (0%)	1 (2%)	
Medication				n.s.
SSRI/SNRI, n (%)		6 (30%)	22 (41%)	
Stimulants, n (%)		6 (30%)	13 (24%)	
Antipsychotics, n (%)		0 (0%)	2 (4%)	
Melatonin, n (%)		5 (25%)	17 (31%)	
Antihistamine, n (%)		2 (10%)	8 (15%)	

Abbreviations: CY-BOCS = Children's Yale-Brown Obsessive Compulsive Scale; CGI-S = Clinical Global Impression – Severity; ADHD = Attention Deficit Hyperactivity Disorder; BDD = Body Dysmorphic Disorder; SSRI = Selective Serotonin Reuptake Inhibitors; SNRI = Serotonin–Norepinephrine reuptake inhibitors.

### 3.2 Primary outcome

**Fig. 2:**
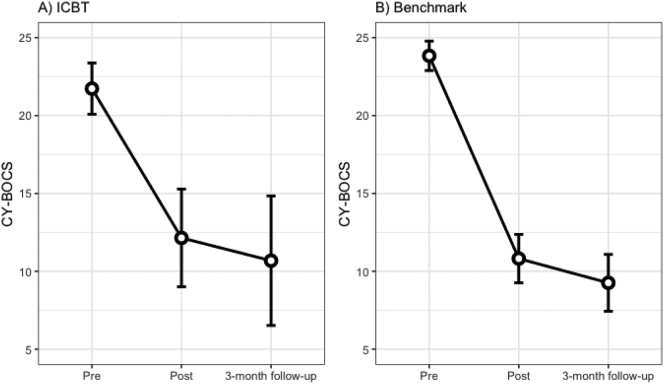
Estimated mean values and 95% confidence intervals at pre-treatment, post-treatment and 3-month follow-up for (A) ICBT sample and (B) benchmark sample.

In the ICBT sample, CY-BOCS scores decreased from M = 21.7 (95% CI[20.2–23.2]) at pre-treatment to M = 12.2 (95% CI[9.6–14.8]) at post-treatment, corresponding to a statistically significant change (β = 9.59, *t* = 6.98, *p* < .0001). There was no additional change between post-treatment and follow-up (β = 1.46, *t* = 0.80, *p* = .71) (Figure 2a). The change from pre-treatment to the 3-month follow-up corresponded to a large within-group effect of *d* = 1.33 (95% CI[0.74–1.92]).

In the benchmark sample, there was a significant decrease on the CY-BOCS from M = 23.8 (95% CI[22.9–24.8]) at pre-treatment to M = 10.8 (95% CI[9.2–12.5]) at post-treatment (β = 13.02, *t* = 16.82, *p* < .0001) and no additional improvement from post-treatment to the 3-month follow-up (β = 1.56, *t* = 2.02, *p* = .11) (Figure 2b). The pre-treatment to 3-month follow-up difference on the CY-BOCS corresponded to a large within-group effect size of *d* = 2.69 (95% CI[1.94–3.45]).

### 3.3 Treatment response and remission

In the ICBT sample, 52% of participants were classified as responders at post-treatment, further increasing to 61% at follow-up (non-significant change between timepoints, *p* = .99). Regarding remission, there were 43% remitters at post-treatment and 50% at follow-up (non-significant change, *p* = .99).

In the benchmark sample, the proportion of responders was 78% at post-treatment and 70% at follow-up (non-significant change between timepoints, *p* = .34). Regarding remission, there were 54% fulfilling remission criteria at post-treatment and 61% at follow-up (non-significant change, *p* = .42).

### 3.4 Self- and parent-rated measures

In both samples, secondary measures improved from pre-treatment to post-treatment and were maintained at 3-month follow-up. Effect sizes were medium-to-large in the ICBT sample. In the benchmark sample, effect sizes ranged from small to large. For detailed results, see Table 2.

**Table 2:** Estimated means (95% CI) for self- and parent-rated measures at pre-, post-treatment and 3-month follow-up in the ICBT and benchmark samples.

	ICBT sample						Benchmark sample					
	Pre	Post	p^a^	3 months	p^b^	d^c^	Pre	Post	p^a^	3 months	p^b^	d^c^
Self-rated												
OCI-CV	18.6 (15.5–21.7)	12.1 (8.6–15.7)	<0.001	12.7 (9.2–16.2)	0.99	0.87 (0.33–1.40)	17.8 (15.8–19.9)	11.7 (9.5–14.0)	<0.0001	12.4 (10.3–14.5)	0.96	0.76 (0.38–1.13)
SMFQ-C	10.9 (8.2–13.5)	6.3 (3.3–9.2)	0.003	6.8 (3.8–9.8)	0.98	0.66 (0.24–1.07)	11.7 (9.4–14.1)	7.6 (5.2–9.9)	0.003	8.7 (6.3–11.0)	0.54	0.24 (−0.06–0.54)
WSAS-Y/C	17.6 (14.0–21.2)	8.2 (4.6–11.9)	<0.0001	11.6 (7.1–16.0)	0.52	0.58 (−0.03–1.19)	17.5 (15.2–19.9)	9.5 (7.3–11.8)	<0.0001	8.9 (6.3–11.6)	0.99	1.04 (0.63–1.46)
Parent-rated												
FAS-SR	28.5 (20.3–36.7)	12.5 (6.2–18.7)	<0.001	15.8 (8.2–23.4)	0.81	0.64 (0.17–1.10)	28.4 (23.2–33.7)	11.1 (7.0–15.2)	<0.0001	12.3 (7.6–17.0)	0.99	0.91 (0.55–1.28)
SMFQ-P	10.2 (7.9–12.5)	6.6 (4.4–8.9)	0.03	6.7 (4.1–9.3)	1.0	0.74 (0.14–1.34)	11.8 (10.4–13.3)	7.68 (6.2–9.2)	<0.0001	7.0 (5.4–8.6)	0.90	0.86 (0.50–1.22)
WSAS-Y/P	23.2 (20.0–26.4)	14.6 (10.7–18.4)	<0.0001	14.9 (10.1–19.7)	0.99	0.71 (0.34–1.09)	23.8 (21.7–25.8)	13.0 (10.5–15.4)	<0.0001	13.8 (10.9–16.8)	0.95	1.21 (0.79–1.64)

a = *p*-values for the pre-treatment to post-treatment comparison; b = *p*-values for the pre-treatment to 3-month follow-up comparison; c = Cohen's d for the pre-treatment to 3-month follow-up comparison. Abbreviations: ICBT = Internet-delivered Cognitive Behavior Therapy; OCI-CV = Obsessional Compulsive Inventory – Child Version; SMFQ-C or P = Mood and Feelings Questionnaire, Child or Parent version; WSAS-Y/C or P = Work and Social Adjustment Scale – Child or Parent version; FAS-SR = Family Accommodation Scale – Self-Report.

### 3.5 Post hoc benchmarking analysis

The benchmarking analysis showed that the difference of the pre-treatment to 3-month follow-up effect sizes of the two samples was significantly larger than the predefined maximal difference for equivalence (Δ = 0.2), indicating non-equivalence of the two treatments (*p* = .003).

### 3.6 Number of sessions, therapist time, adherence, credibility and patient satisfaction

Out of the 14 online treatment modules the patients completed on average 6.41 (SD = 4.00) modules and the parents completed on average 7.50 (SD = 4.53) modules. The average treatment time for the therapists in the ICBT group was 3 h and 15 min (SD = 2 h 25 min) per patient for the entire treatment. The mean number of in-person sessions in the benchmark group was M = 14.52 (SD =5.79), corresponding to an approximate total of at least 28 h per patient.

The mean score on therapist-rated adherence for the first half of the ICBT treatment (iiPAS-mid) was M = 8.90 (SD = 5.05) and M = 5.19 (SD = 4.47) for the second half of the treatment (iiPAS-post), indicating significantly less adherence in the second half of ICBT (*t* = 3.24, *p* = .004). Patients in ICBT rated the credibility of the treatment on average M = 33.95 (SD = 8.24, possible max score on the credibility measure is 50). A credibility rating was not available for the benchmark sample.

The child CSQ patient satisfaction index was on average M = 22.3 (SD = 4.81) in the ICBT group and M = 26.7 (SD = 3.35) in the benchmark group. The parent CSQ satisfaction ratings were M = 25.4 (SD = 3.82) and M = 29.2 (SD = 2.60), respectively. Patient and parent satisfaction ratings were negatively correlated with the CY-BOCS total score at the post-treatment time point in both groups (correlation coefficients ranging from *r* = −0.40 to −0.57; all *p*-values statistically significant, except for the parent ratings in the ICBT group).

### 3.7 Parent and clinician feedback

Participating children and their parents reported the ICBT program to be accessible and allowing flexibility. The content was experienced as helpful and informative and the format was said to encourage the child's independence. Some children experienced the treatment content as too extensive, causing boredom and concentration issues. Some parents reported difficulties to motivate their children to engage regularly with the treatment. Some children did not feel represented by the case vignettes presented when their particular type of OCD was not exemplified. Some families wished for more support in real time, such as telephone calls or in-person meetings.

The involved therapists considered the ICBT program to be helpful for those able to comprehend the treatment rationale with minimal support. Therapists also appreciated that the ICBT program brought structure to the treatment and helped to keep the intervention focus on the OCD. Therapists also noted that those who had executive functioning problems and negative experiences of previous CBT treatment had more problems adhering to ICBT.

## 4 Discussion

We evaluated the feasibility of an adapted ICBT protocol for OCD in young people with ASD. The main finding was that ICBT was deemed acceptable by the patients and their families and was associated with clinically significant improvements in OCD symptom severity, which were sustained at the 3-month follow-up (Cohen's *d* = 1.33). Similarly, significant improvements were observed in most of the secondary parent- and self-rated measures. Approximately 60% of the participants were classed as treatment responders and 50% were in remission from their OCD at the 3-month follow-up. These outcomes are slightly better than those obtained in the largest naturalistic study of children and adolescents with ASD and OCD (*N* = 172) who were treated in-person at a specialist clinic in the UK (Jassi et al., 2021). In that study, 53% of the participants were classed as treatment responders and 31% as being in remission. However, it is likely that the participants in that study were more severe at baseline.

To provide a meaningful benchmark, we also analyzed data from a specialist clinic that regularly treats individuals with this comorbidity using conventional in-person treatment. Individuals in the benchmark sample experienced robust symptom reductions (Cohen's *d* = 2.69) and a higher proportion (70%) were deemed responders and being in remission (61%) at the 3-month follow-up. These results were further confirmed by a post hoc benchmark analysis (Minami et al., 2008), showing that ICBT was not equivalent to the benchmark treatment. These findings were largely expected, as there were some important differences between the two samples. First, whereas ICBT participants specifically volunteered for the study, the benchmark sample were regular clinic patients who consented for their routinely collected data to be used for research. Second, the ICBT sample had additional exclusion criteria (medication changes not allowed), whereas no such restriction was imposed on the benchmark sample. Third, patients in the benchmark group had nearly unlimited access to therapists at a highly specialized unit for OCD and related disorders, including home visits when needed. Thus, we urge caution in the interpretation of these benchmark analyses. A formal head-to-head comparison with identical treatment protocols would be required to fully evaluate the relative efficacy of ICBT vs in-person CBT for OCD in young people with ASD.

From a health economic perspective, there were striking differences in the amount of therapist input required by ICBT participants vs benchmark participants. On average, ICBT participants required 3 h and 15 min of therapist time for the entire study period. By contrast, patients in the benchmark group had received on average 14 in-person sessions and required multiple therapists; this would translate to a minimum of 28 h of therapist time for each patient (a conservative estimate). Thus, ICBT is likely a cost-effective and resource-efficient intervention for this patient group.

Our adapted ICBT protocol was deemed acceptable to the participating families and therapists. The majority of participants completed treatment. Interestingly, participants and their parents completed approximately six to seven of 14 possible online modules. By contrast, participants in our recent OCD trial, which excluded individuals with ASD, completed ten to eleven out of the 14 available modules (Aspvall and Andersson, 2021). Even though the adherence to ICBT in the current study was sufficient to result in clinically meaningful symptom improvements, the module completion rates may be sub-optimal and point towards a need to further adapt ICBT to the cognitive and motivational needs of young persons with ASD. Patient and parent satisfaction were inversely and substantially correlated with OCD symptom severity at post-treatment, suggesting that clinical improvement was the main driver of participant satisfaction.

The study had some limitations that affect the generalizability of the results. Because ASD was assessed and diagnosed by other services prior to participation in the study, reliable quantitative data regarding ASD symptom severity was not available for either of the two samples. The non-randomized design of the study meant that the two samples originated from slightly different populations. For example, the amount and intensity of treatment received was much greater in the benchmark sample. Thus, any between group comparisons, including our benchmarking analyses, should be interpreted with great caution. Finally, the study was conducted at a highly specialized OCD clinic with experienced and well-trained clinicians in the field of OCD and ASD. The results may therefore not translate to other clinical contexts, such as primary care or non-specialist CAMHS services.

## 5 Conclusions

To conclude, ICBT can be successfully adapted to treat OCD in youth with ASD. Further improvements of the treatment protocol based on participant and therapist feedback are warranted, as is a formal test of its efficacy in a randomized controlled trial.

## Funding

This project was funded by 10.13039/501100006350Stiftelsen Clas Groschinskys Minnesfond, Stockholm, Sweden, and Centre for Psychiatry Research, Department of Clinical Neuroscience, Karolinska Institutet and Stockholm Health Care Services, Region Stockholm.

## Declaration of competing interest

None.
